# Supplementary material for: Immunogenicity of an Inactivated Senecavirus A Vaccine with a Contemporary Brazilian Strain in Mice
Source: Vaccines (Basel). 2024 Jul 26;12(8):845. doi: 10.3390/vaccines12080845 (PMC11358955; doi:10.3390/vaccines12080845)
Supplement: Supplementary file 1 [file vaccines-12-00845-s001.zip › vaccines-3059862-supplementary.pdf]

# Supplementary material

Immunogenicity of an inactivated Senecavirus A vaccine with a contemporary Brazilian strain in mice

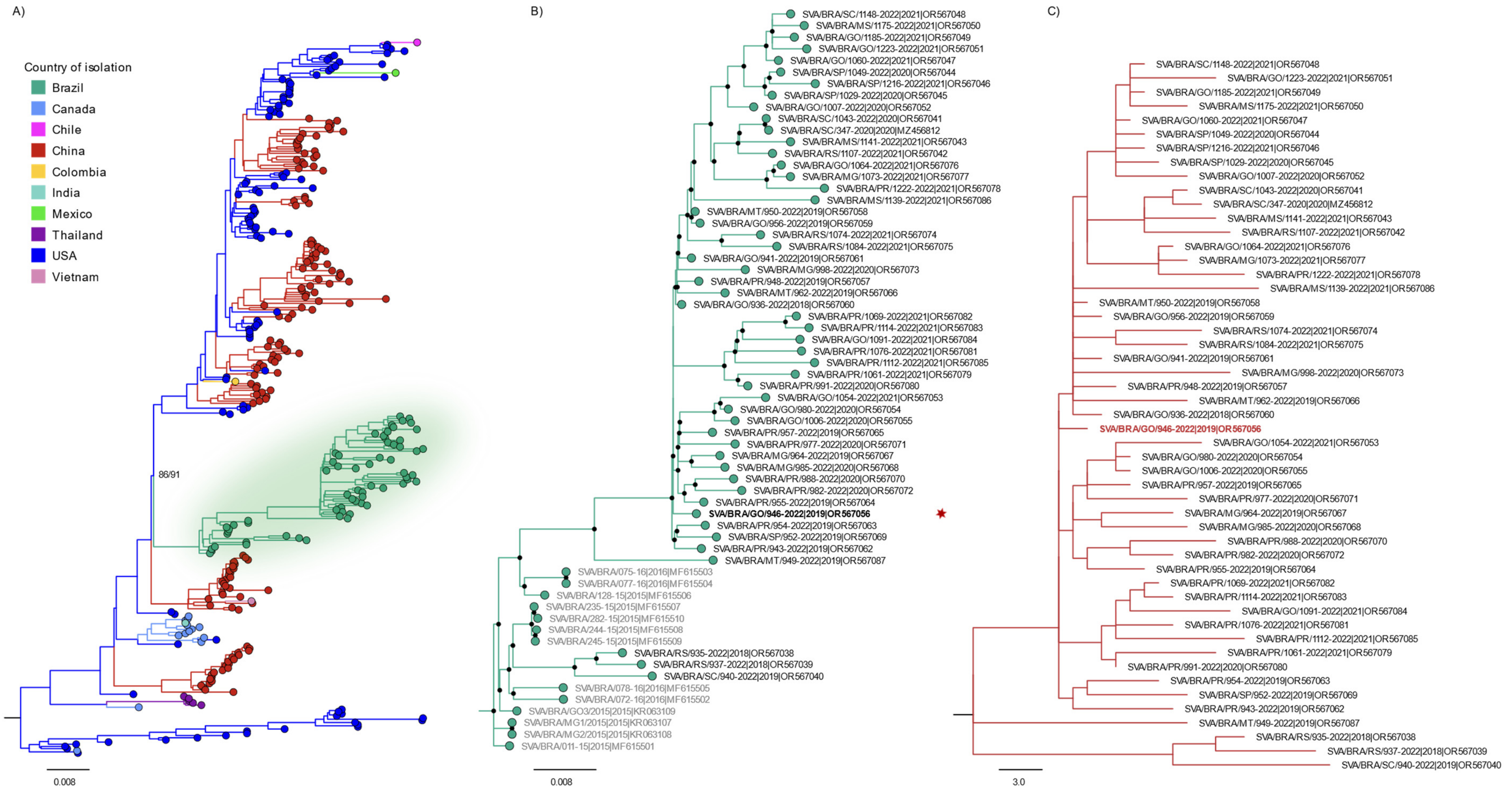

**Figure S1.** ML phylogenetic tree inferred for 328 full-ORF SVA sequences collected globally from 1988 to 2022. A) Phylogenetic tree showing that Brazilian strains grouped separately from other countries' isolates, forming a statistically supported monophyletic clade. Branches are shaded by country of origin according to the figure key. The tree is midpoint-rooted for clarity. Branch support values are provided for key nodes. B) Topology details of the Brazilian clade containing all SVA sequences until the time of this study. Strains collected from 2018 to 2021 are colored black; older strains (before 2018) are colored gray. The strain selected as the best representative of Brazilian SVA is indicated by a red star. Black circles at the internal nodes indicate clades supported by SH-aLRT >= 80% and UFB >= 95%. C) The output tree generated by PARNAS algorithm. The sequence chosen as the best representative covers 98% of the amino acid genetic diversity observed in the Brazilian strains isolated during 2018-2021. The amino acid sequence alignment (SVA polyprotein) was included in the analysis. The tree was re-scaled to reflect the number of amino acid substitutions along the branches.

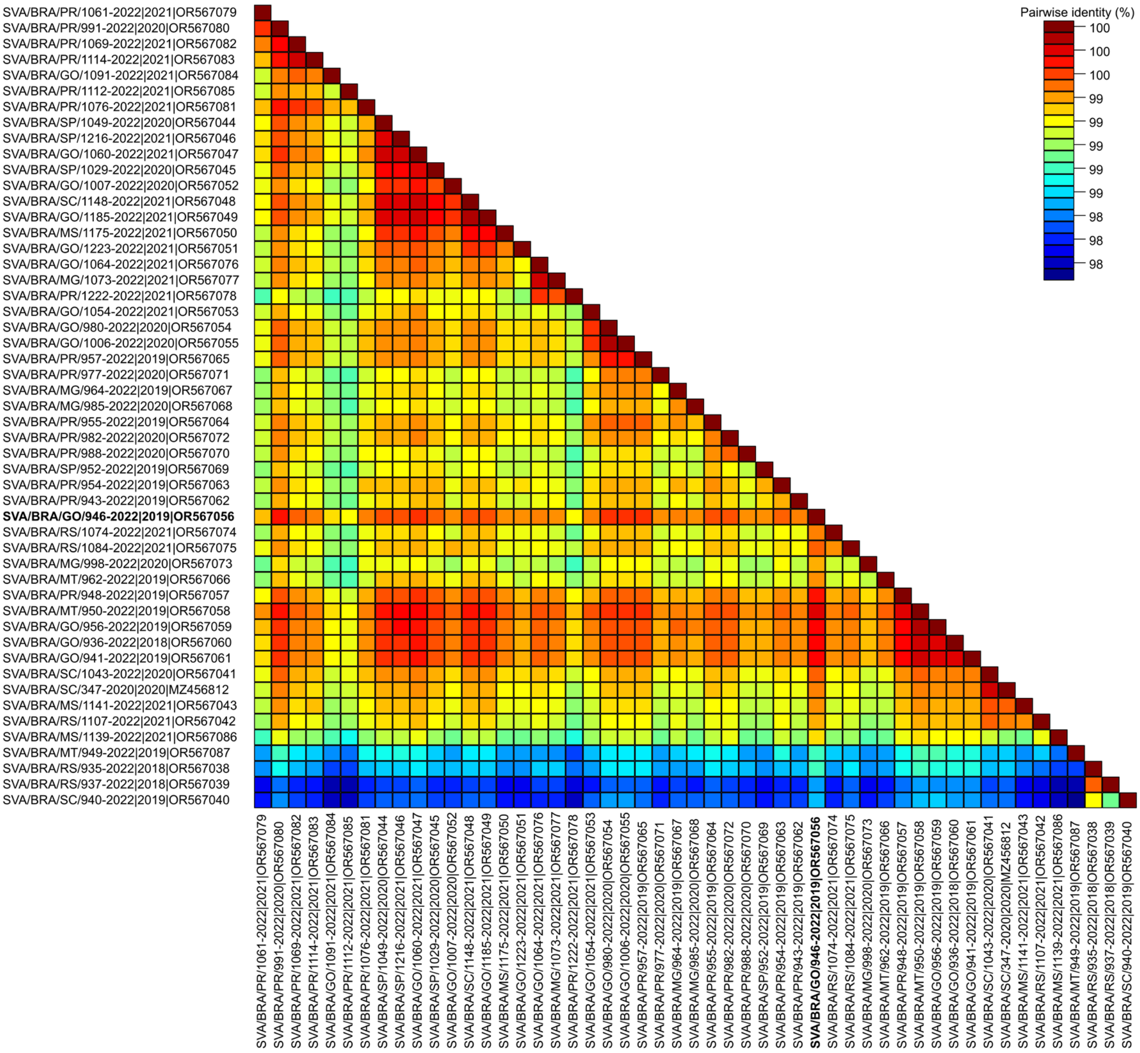


**Figure S2.** Pairwise identity matrix based on the amino acid sequences of full-length ORF from 51 SVAs isolated in Brazil during 2018- 2021. The strain selected for the vaccine formulation is highlighted in bold.

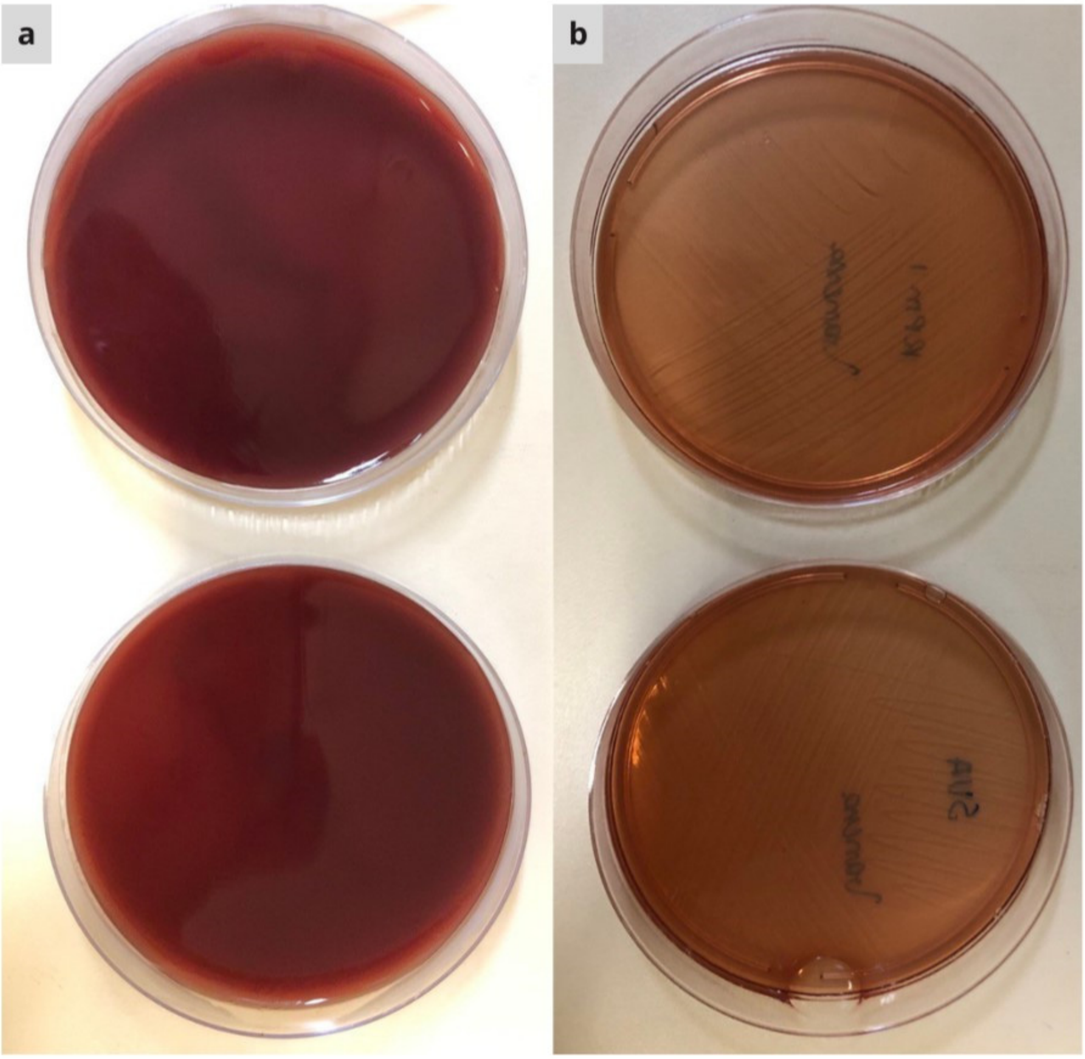


**Figure S3.** a- Blood agar seeding with the inactivated viral suspension showing no bacterial growth after 24 hours of incubation at 37 ºC. b- MacConkey agar seeding with the inactivated viral suspension showing no bacterial growth after 24 hours of incubation at 37 ºC.

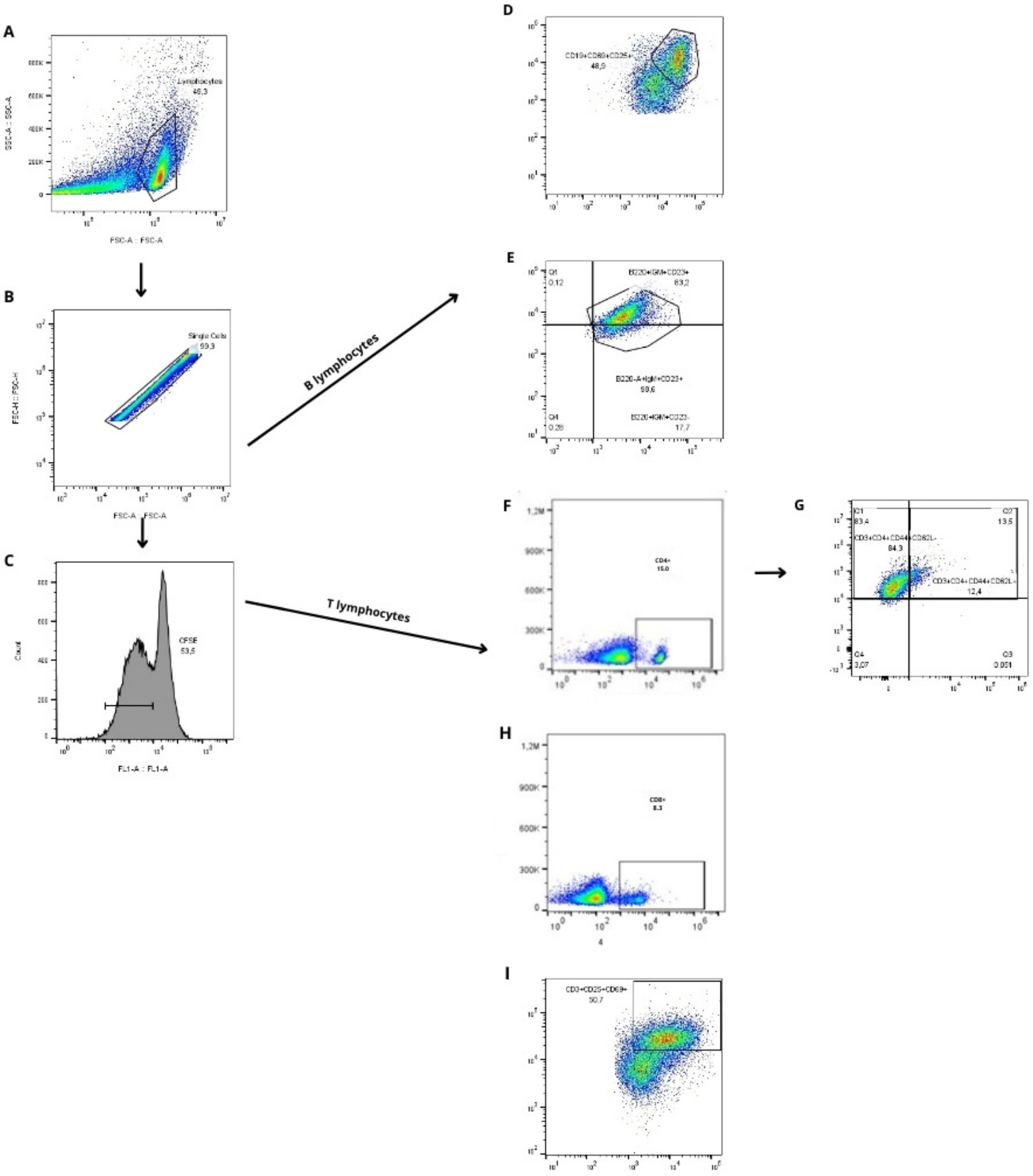


**Figure S4.** A- Forward scatter (FSC) vs. side scatter (SSC) plot to identify the lymphocyte population. B- FSC-H vs. FSC-A plot to exclude doublets. C- Flow cytometry histograms gated into CFSE+ events show a low CFSE intensity, indicating lymphocyte proliferation. Multiple plots show gating strategies for various B (D and E) and T cell subsets (F, G, H and I), including CD19+CD69+CD25+ (D), B220+IgM+CD23+ (E), CD4+(F), CD3e+CD4+CD44+CD62L+ and CD3+CD4+CD44+CD62L- (G), CD8α+(H) and CD3+CD25+CD69+(I).
